# Supplementation of the diet with the functional fiber PolyGlycoplex^® ^is well tolerated by healthy subjects in a clinical trial

**DOI:** 10.1186/1475-2891-8-9

**Published:** 2009-02-05

**Authors:** Ioana G Carabin, Michael R Lyon, Simon Wood, Xavier Pelletier, Yves Donazzolo, George A Burdock

**Affiliations:** 1 Burdock Group, Orlando, Florida, USA; 2 Canadian Centre for Functional Medicine, Coquitlam, British Columbia, Canada; 3 Food, Nutrition and Health Program, University of British Columbia, Coquitlam, British Columbia, Canada; 4 OPTIMED Clinical Research, Gieres, France

## Abstract

**Background:**

The relationship of dietary fiber to overall health is of great importance, as beneficial effects have been demonstrated with the use of fiber from diverse sources, some traditional, other novel. PolyGlycopleX^® ^(PGX^®^) is a unique proprietary product composed of three water-soluble polysaccharides, that when processed using novel technology give rise to a final product – a soluble, highly viscous *functional fiber*.

**Methods:**

Because of its potential use in food and dietary supplements, a randomized, double-blind, placebo controlled clinical study was conducted to evaluate the tolerance to PGX ingestion for 21 days, to a maximum dose level of 10 g per day, in healthy male and female volunteers. The main objective of the study was to evaluate the overall gastrointestinal (GI) tolerance, while secondary objectives were to evaluate possible changes in hematological, biochemical, urinary and fecal parameters.

**Results:**

Results show that PGX is well tolerated as part of a regular diet with only mild to moderate adverse effects, similar to those seen with a moderate intake of dietary fiber in general, and fruits and vegetables. Because PGX is a highly viscous, *functional fiber*, it also demonstrates several physiological responses including, but not limited to maintaining healthy total and LDL cholesterol and uric acid levels.

## Background

The Institute of Medicine (IOM) report [1] on dietary fiber recognizes the importance of fiber to overall health and acknowledges the diversity of edible, non-digestible carbohydrates in the human food supply, while allowing for flexibility to incorporate new fiber sources developed in the future. In 2002, the Food and Nutrition Board of the Institute of Medicine published the report on fiber, introducing new definitions and classifications, and discerning between fiber found naturally in plants (*dietary fiber*; *e.g*., cellulose, pectin, gums, hemicellulose, β-glucans, and fiber contained in oat and wheat bran), and isolated or synthetic fiber that may be used in dietary supplements or added to foods (potential *functional fiber *for food labeling; isolated (*e.g*., resistant starch, pectin, and gums), animal, or commercially produced carbohydrates) [1]. The new IOM definition for *total fiber *is "the sum of *dietary fiber *and *functional fiber*", attempting to encompass both the physical characteristics and the physiological effects of fiber in humans [1].

Dietary fibers display a wide range with regards to viscosity. Certain dietary fibers including, but not limited to pectins, *beta*-glucans, psyllium, and gums have unique physicochemical properties that allows them to form viscous solutions or gels when mixed with liquids, the degree of thickening being directly dependent on the chemical composition [2,3].

Gums consist of a diverse group of polysaccharides. These hydrophilic polymers have water binding properties and are used in foodstuff to stabilize emulsions, prevent ice recrystallization and impart organoleptic properties. Several gums are approved by the Food and Drug Administration (FDA) for their technical functional effects as stabilizers and thickeners (21 Code of Federal Regulations (CFR) §170(o)(28)).

One physicochemical property of fiber, viscosity, has been investigated and found to affect several possible physiological responses [4,5]. In general, when fiber is mixed with food and human digesta in the gut, a firm soluble polysaccharide/food matrix is formed leading to delay of gastric emptying. Fiber, and especially viscous fiber will result in a sensation of fullness, and possible alterations in blood glucose and cholesterol concentrations, and slower transit time through the small intestine [4,5].

Dietary fibers have also been classified based on solubility and fermentability. Soluble dietary fibers including *beta*-glucans, mucilages (*e.g*., psyllium), pectins, some hemicelluloses and gums, are dispersible in water. Pectins, *beta*-glucans, gums, inulin and oligofructose are dietary fibers that are also readily fermented by bacteria in the colon leading to the formation of short chain fatty acids (primarily acetate, propionate, and butyrate) and gases (carbon dioxide, methane, hydrogen) [1,6-8].

PolyGlycopleX (PGX) (PolyGlycopleX^® ^and PGX^® ^are registered trade marks) is a proprietary product manufactured with the use of novel technology under good manufacturing practices (GMP). The product is composed of three highly-purified, water-soluble polysaccharides: konjac powder, sodium alginate, and xanthan gum. The three gums act synergistically to form strong bonds that lead to a level of viscosity that is 3 to 5 times higher than any known individual polysaccharide. The final product is a soluble highly viscous *functional fiber *that can be used to provide consumers with a supplementary source of fiber in the diet. The present clinical study was designed to evaluate the tolerance to PGX ingestion for 21 days, to a maximum dose level of 10 g per day, in healthy male and female volunteers.

In pre-clinical testing, PGX was administered in a 90-day feeding study at dietary concentrations of 0, 12,500, 25,000 and 50,000 ppm to male and female Sprague Dawley rats [9]. Results showed no toxicological effects from the use of the test substance in any of the test groups, indicating a no-observed-adverse-effect-level (NOAEL) for PGX at the highest dose tested, corresponding to an average daily intake of 3219 and 3799 mg/kg bw/day in male and female rats, respectively [9].

## Methods

### Objectives

The main objective of the study was to evaluate the overall gastrointestinal (GI) tolerance to the fiber, while secondary objectives were to evaluate GI symptoms and signs (clinical examination) upon repeated consumption of PGX by healthy men and women for 21 days, and assess any effects on blood biochemical, hematological, urinary and fecal analysis.

### Subjects and methods

#### Study population

Eighty-eight volunteers were screened for the study. Following selection, 54 healthy subjects, 25 (46.3%) males and 29 (53.7%) females, ranging in age from 18 to 55 (mean age 31.6 ± 10.5 years) entered the clinical trial. All subjects were deemed healthy, based on physical examination (including heart rate, blood pressure, auscultation of heart and lungs, and abdominal palpation and auscultation), electrocardiogram (ECG) and laboratory tests that were within normal limits. The height of the subjects ranged from 155 cm to 191 cm (mean 170.9 ± 8.3 cm), the weight ranged from 48.5 to 88.6 kg (mean 66.5 ± 8.6 kg), and the calculated body mass index (BMI) ranged from 18.4 to 28.4 kg/m^2 ^(mean 22.7 ± 2.2 kg/m^2^).

Subjects were randomly assigned to one of two groups: study group (PGX) (14 males and 13 females), and control group (skim milk powder) (11 males and 16 females). All 54 subjects completed the clinical study.

#### Inclusion/Exclusion criteria

Included in the study were non-smoking (or smoking less than five cigarettes *per *day), healthy (based on physical examination, medical history, ECG and laboratory tests), males and females, 18 to 55 years of age, with a BMI between 18.5 and 29.9 kg/m^2^, and a stable weight for the prior two months, agreeing to eat cereals and yogurt, 2 times *per *day as part of two out of three main meals, for 3 consecutive weeks.

Exclusion criteria used for the study are found in Table 1. Subjects were allowed to withdraw from the study at any time and irrespective of reason, or because of a serious adverse event.

**Table 1:** Exclusion criteria

Abnormal physical examination
History of GI disease (i.e., gastric ulcers, irritable bowel disease, history of bowel obstruction and colon cancer)
History of abdominal surgery (exception: appendectomy)
Pregnant or beast feeding women
BMI > 30 kg/m^2^
Metabolic disorders (i.e., diabetes, and metabolic syndrome)
Using prescription medications, H_2 _blockers*, anti-acids, OTC**, dietary & herbal supplements
Involved in a weight loss program
Being treated for eating disorders
Having participated in another clinical trial in the previous month
Having received general anesthesia in the previous month
Refusing to consume the foods provided in the study
Known allergies to milk, nuts, wheat, soy, oat, and barely
Refusal to sign consent

*H_2 _blockers, also known as histamine-2 receptor antagonists, are drugs that prevent or block the production of gastric acid.

**OTC – over-the-counter

#### Study design

The clinical trial was randomized, double-blind, placebo-controlled, lasting three weeks, in addition to a two-week enrollment period. The outpatient study was conducted at a single research facility in accordance with the Declaration of Helsinki (Edinburgh, 2000), and following Good Clinical Practice Guidance (ICH E6), and local (French) regulatory requirements. The protocol was approved prior to study initiation by the French ethics committee (Comité de Protection des Personnes (CPP) Sud-Est III) and health authorities (DGS). There were no major protocol deviations identified during the study. Written informed consent was obtained from all subjects prior to study commencement.

#### Study products

The test product used in the study was PolyGlycopleX (PGX) supplied by the sponsor (Inovobiologic; Calgary, Alberta Canada). Two and a half grams of PGX were ingested BID (L. *bis in die*, or twice a day) as part of two main meals (breakfast and/or lunch, and/or dinner), for the first seven days, followed by 5 g PGX BID for the last 14 days of the study. The control product selected for use in the study, based on color and orosensory (texture similarity) match to the test product was a skimmed milk powder taken as 2.5 g BID for one week and then 5 g BID for the remainder of the study. The composition of the control product included 35.5% proteins, 51.7% carbohydrates, 0.80% lipids, 1.29% calcium, 0.11% magnesium and 0.95% phosphorous.

Test and control products (2.5 g) were pre-mixed with 10 g breakfast cereals (Extra Pépites, Kellogg's^®^) (the amount of fiber supplied by the cereal portion was 0.45 g) by CRID Pharma, France. Each container was then packaged with Le yogourt nature (plain) de Danone, a commercially available yogurt (135 ml); the pre-mixed product and yogurt were combined by each subject prior to ingestion. Test and control products were packaged individually for each subject, as ready-to-use portions, and were supplied in strictly identical neutral packaging, making it impossible for subjects and investigators to distinguish one from the other, therefore ensuring the double blinding for the study. The products were packaged and released by CRID Pharma, France.

#### Selection of doses for the study

The tolerance of PGX was tested compared to the literature values established for other similar carbohydrates (*e.g*., fiber). Therefore, it was decided to evaluate the tolerance to ingestion of PGX for up to 10 g *per *day.

#### Study parameters

The parameters assessed during the clinical trial included GI discomfort (intensity of abdominal pain and nausea, flatulence, vomiting, intestinal rumbling, bloating and pain, stool frequency and consistency), plasma chemistry, hematology, urine, feces and plasma vitamin levels. The biochemical parameters analyzed included glucose, sodium, potassium, chloride, magnesium, calcium, zinc, creatinine, urea, uric acid, total protein, albumin, total bilirubin, alkaline phosphatase, aspartate transaminase (AST; or aspartate aminotransferase), alanine transaminase (ALT; or alanine aminotrasferease), gamma-glutamyl transpeptidase (GGTP; or gamma-glutamyl transferase (GGT)), lactate dehydrogenase (LDH), creatinine phosphokinase (CPK), total cholesterol, high-density lipoprotein (HDL) cholesterol, low-density lipoprotein (LDL) cholesterol, triglycerides, ferritin, and thyroid stimulating hormone (TSH). Hematological testing included red blood cells, hematocrit, hemoglobin, mean corpuscular hemoglobin, mean corpuscular hemoglobin concentration, mean corpuscular volume, leucocytes, neutrophils, eosinophils, basophils, lymphocytes, monocytes and platelets. Urinalysis tested the pH, glucose, proteins, blood, and ketones, while stool analysis tested the pH, wet and dry matter, and short chain fatty acids (SCFA; I-butyrate, N-butyrate and the ratio of butyrate/(acetate + propionate). Plasma levels for vitamins A, B_1_, B_6_, B_12_, C, 1,25 OH vitamin D, E, and K were also tested during the study.

Biochemical analyses were performed on the multi-parametric automated system Roche/HITACHI 912, Roche Diagnostics, with a bi-directional connection. All parameters were analysed using commercially available kits. No adaptation of the commercial methods was made. Methods of analysis are found in Appendix I [see additional file 1].

#### Visits

Physical examinations were performed on all subjects at V0, V2 and V3 visits, and were all within normal limits.

Throughout the duration of the study, the clinical condition of each subject was assessed during four separate visits: V0 (screening); V1 (baseline visit), V2 (following one week of consumption of 5 g *per *day of the assigned product), and V3 (following two weeks of consumption of 10 g *per *day of the assigned product).

Screening (V0) took place between Day -15 and Day -1, at which time the informed consent was reviewed with each volunteer and signed. Each subject underwent a physical examination including recording of the height, weight, heart rate, orthostatic blood pressure (5 minute supine and 1 minute standing), auscultation of the heart and lungs, and palpation and auscultation of the abdomen for bowel sounds.

At baseline visit (V1; Day 0), subjects were randomized to either test or control group, and underwent blood and stool sampling. Volunteers received diaries and pre-packaged products that included 2.5 g of the test or control product to be consumed BID for 7 days.

At the visit following one week (V2; Day 8 ± 1) of consumption of 5 g of the test or control product *per *day, the subjects underwent evaluation of clinical tolerance following the use of 2.5 g test or control products BID, and provided additional blood and stool samples. The subjects turned in their diaries and empty product containers, or unused product, and were provided with new diaries and pre-packaged products for the remainder of the study. They were instructed to consume 5 g of the test or control product, BID for 14 days.

At the visit following two weeks (V3; Day 22 ± 2) of consumption of 10 g of the test or control product *per *day, the subjects underwent evaluation of clinical tolerance following the use of 5 g test or control products BID, and provided additional blood and stool samples. The subjects turned in their diaries and empty product containers, or unused product.

#### Diet

Subjects were asked to abstain from consuming fiber-rich foods, and to adhere to a low-fiber diet of approximately 10 g *per *day.

#### Evaluation of GI signs and symptoms

The main assessment criteria were conducted using self evaluation of GI discomfort during the preceding week as reported by the subject at each visit, while the secondary criteria were performed using weekly mean evaluation of daily values of intestinal symptoms, clinical evaluation by the investigator, and laboratory analysis results (blood chemistry and stool analysis). Subjects recorded the intake of test and control product and the presence and intensity of digestive symptoms in individual diaries, which were handed in at subsequent visit. The recorded parameters and their intensity were based on each subjects' self evaluation of the stool frequency, stool consistency, and GI discomfort (flatulence, borborygmus, meteorism, abdominal pain, nausea, and vomiting). The same parameters were also evaluated at the V2 and V3 visits through the use of Visual Analog Scale (VAS is "a testing technique for measuring subjective or behavioral phenomena (as pain or dietary consumption) in which a subject selects from a gradient of alternatives (as from "no pain" to "worst imaginable pain" or from "every day" to "never") arranged in linear fashion") [10]. The VAS scale used in the study was from 0 (none) to 100 (very intense) and was completed by each subject in the presence of the investigator. Additionally, clinical examinations of individual subjects were conducted at each visit by the investigator that included, but was not limited to palpation of the abdomen for signs of discomfort or tenderness, and auscultation of the abdomen for hypo- or hyperactive bowel sounds.

Signs and symptoms were recorded through out the study and their occurrences analyzed statistically. *Signs *were defined as any abnormality indicative of disease, discoverable on examination of the patient; an objective symptom of disease, in contrast to a *symptom *which is a subjective sign of disease [11].

The frequency and intensity of signs and symptoms determined the threshold of GI tolerance.

#### Compliance

Compliance with the instructions provided at commencement of the study was assessed throughout the study. At each visit, subjects were asked to turn in their diaries, and at V2 and V3 each subject was required to bring back all used packaging and unused product. The following formula was used to determine compliance:

% compliance = number of products taken/theoretical number of products to be taken × 100

#### Stool collection

Forty eight hours prior to each visit, subjects were asked to collect one stool sample in designated containers. Stool samples were dropped off by each subject at the study site within twelve hours of collection, and were stored at 5 ± 3°C until analysis, which was conducted using standard laboratory methods described in the protocol. The time from collection of the stool samples to analysis was generally 24 hours and never exceeded 36 hours.

#### Concomitant treatments

No concomitant drug treatment was allowed during the study. However, under special circumstances, and properly documented in the case report form (CRF) the occasional use of paracetamol was allowed, as well as that of prescription medication when approved by the investigator. Depending on the event and medication used, re-evaluation of the continued participation of the subject in the study was assessed and shared immediately with the sponsor, especially in case of prolonged use of oral antibiotics.

### Statistical analysis

The statistical analysis plan was written, validated and included in the protocol, while the statistical analysis was conducted at the conclusion of the clinical study. All individual data for all the subjects in the study were presented in data listings, sorted by group of product, subject, and visit. Demographic and baseline characteristics data were summarized by group of product and overall subjects. Demographic characteristics (sex, age, weight, height, and BMI), vital signs (blood pressure and heart rate), biology (biochemistry, plasma vitamin levels, hematology, and urinalysis), stool analysis, self evaluation parameters by the subjects: stool frequency, stool consistency, GI discomfort symptoms (flatulence, borborygmus, meteorism, abdominal pain, nausea, and vomiting), and clinical evaluation of abdominal signs, were compared between test and control groups using analysis of variance or Mann-Whitney-Wilcoxon test for quantitative parameters, using chi-2 test or Fisher's exact test for qualitative parameters. In the case that normality of distribution of the main criterion was not respected, the main criterion was compared using Mann-Whitney-Wilcoxon test (with *t *approximation) on value and change from baseline of parameter at V3. Test and control products were compared using ANOVA or Wilcoxon test, depending on the normality of the distribution. The normality of distribution was tested by a Shapiro-Wilk's test.

## Results

Fifty four healthy volunteers (25 males and 29 females), ranging in age between 18 and 55 years, entered into and completed a three-week study devoted to assessing the tolerance of PGX when ingested twice daily for three weeks as part of a low-fiber diet to a maximum dose level of 10 g *per *day. The subjects' baseline clinical and biochemical characteristics were not statistically different. Statistical analysis of VAS based on the self evaluation of GI discomfort showed no differences between test and control groups on the intensity of flatulence, intestinal rumbling, bloating, nausea and vomiting at V2 and V3 (Wilcoxon test; p = 0.3907 and p = 0.3722; p = 0.6360 and p = 0.8161; and p = 0.2402 and p = 0.1251; p = 0.8251 and p = 0.7101; p = 0.8945 and p = 0.9169, respectively), or on the intensity of abdominal pain at V2 and V3 (Wilcoxon test; p = 0.2945 and p = 0.1923, respectively) during the same visits.

Compliance with the instructions provided and intake of the test and control products was assessed throughout the study. The mean average compliance at V2 and V3 (%) for the control group was 100% and for the test group was 99.6%.

The statistical analysis showed no differences between test and control groups on averaged flatulence *per *day, or averaged intensity of borborygmi and bloating *per *day, vomiting, or number of stools at V2 week 1, or at V3 weeks 2 + 3, V3 week 2 or V3 week 3. There was no difference between test and control group on averaged intensity of abdominal pain *per *day at V2 week 1 or at V3 weeks 2 + 3, V3 week 2; however, the averaged intensity of abdominal pain *per *day was statistically significantly higher in the test than control group in V3 week 3 (Wilcoxon test; p = 0.0160). The statistical analysis showed that the average intensity of nausea *per *day was statistically higher in the test than in the control group at V3 week 2 (Wilcoxon test; p = 0.0055), but not at V2 week 1, V3 week 3 and V3 weeks 2 + 3.

At V2, the statistical analysis showed that the consistency of stools was softer in the test than in the control group, although not statistically different (Wilcoxon test; p = 0.0684). However, at V3, there was no difference in stool consistency between the test and control groups (Wilcoxon test; p = 0.3116). The statistical analysis showed no difference between test and control groups on wet stool weight at V2 or V3, or stool pH. However, at visit V2, the *percentage *of dry matter was statistically higher in the control than in the test group (ANOVA; p = 0.0013).

Stool analysis for composition of total short chain fatty acids (SCFA), I-butyrate, N-butyrate and ratio composition of butyrate/(acetate + propionate), showed no product effect between test and control groups on total SCFA, on each SCFA component, or on the ratio of butyrate/(acetate + propionate), at baseline, V2, or V3 visits.

The statistical analysis showed no product effect between test and control groups for each urinary (pH, glucose, proteins, blood, and ketones), or hematological parameter (red blood cells, hematocrit, hemoglobin, mean corpuscular hemoglobin, mean corpuscular hemoglobin concentration, mean corpuscular volume, leucocytes, neutrophils, eosinophils, basophils, lymphocytes, monocytes and platelets) evaluated at baseline, V2, or V3 (data not shown).

The biochemical parameters evaluated at baseline, V2 and V3 included glucose, sodium, potassium, chloride, magnesium, calcium, zinc, creatinine, urea, uric acid, total protein, albumin, total bilirubin, alkaline phosphatase, aspartate transaminase (AST), alanine transaminase (ALT), *gamma*-glutamyl transpeptidase (GGTP), lactate dehydrogenase (LDH), creatinine phosphokinase (CPK), total cholesterol, high-density lipoprotein (HDL) cholesterol, low-density lipoprotein (LDL) cholesterol, triglycerides, ferritin, and thyroid stimulating hormone (TSH). The statistical analysis showed no significant differences between test and control groups for each biochemical parameter evaluated at baseline, V2 or V3, except for total cholesterol, LDL cholesterol, GGTP and uric acid.

The results for fasting total cholesterol (mmol/l), HDL cholesterol (mmol/l), LDL cholesterol (mmol/l) and fasting triglycerides at baseline, V2 and V3 are found in Table 2. The decrease in total cholesterol and LDL cholesterol levels were statistically greater in the test than control group, at visits V2 and V3. A decrease in GGTP and uric acid levels were observed in test versus control groups at V2 and V3, but statistically significant only at V2 (Wilcoxon test; p = 0.0284, ANOVA; p = 0.0316, respectively). At V3, uric acid levels in the PGX and control groups were similar and both were approximately 9% lower than at baseline (Table 3). The statistical analysis showed no significant differences between test and control groups for each plasma vitamin level (A, B_1_, B_6_, B_12_, E, and K) at baseline, V2 or V3, except for vitamin D and C. At visit V2, vitamin D increased level was statistically higher in the test than in the control group (Wilcoxon test; p = 0.0159), while at visit V3, vitamin C was significantly increased over control (ANOVA; p = 0.0324) (Table 4).

**Table 2:** Total, HDL and LDL cholesterol and triglyceride levels at baseline, V2 and V3 visits (N = 54)

		Fasting total cholesterol (mmol/l)		HDL cholesterol (mmol/l)		LDL cholesterol (mmol/l)		Fasting triglycerides (mmol/l)	
	
		Control	PGX	Control	PGX	Control	PGX	Control	PGX
	N	27	27	27	27	27	27	27	27
Baseline	Mean	4.81	4.88	1.75	1.68	2.79	2.89	0.87	0.95
	SD	0.95	0.90	0.44	0.49	0.86	0.73	0.35	0.36
Visit 2	Mean	4.64	4.41	1.70	1.62	2.72	2.59	0.87	0.91
	SD	0.99	0.98	0.39	0.44	0.83	0.68	0.33	0.55
Change at V2 from baseline	Mean	-0.16	-0.47	-0.04	-0.05	-0.07	-0.30	0.00	-0.02
	SD	0.43	0.52	0.19	0.19	0.36	0.43	0.28	0.40
Visit 3	Mean	4.40	4.1	1.63	1.59	2.56	2.41	1.01	0.94
	SD	1.02	0.97	0.41	0.43	0.83	0.70	0.66	0.51
Change at V3 from baseline	Mean	-0.40	-0.70	-0.12	-0.08	-0.23	-0.48	0.14	0.01
	SD	0.53	0.42	0.23	0.21	0.45	0.39	0.58	0.40

HDL = high density lipoprotein; LDL = low density lipoprotein

**Table 3:** Uric acid and *gamma*-glutamyl transpeptidase (GGTP) levels at baseline, V2 and V3 (N = 54)

		Uric acid (μmol/l)		GGTP (IU/l)	
		
		Control	PGX	Control	PGX
	N	27	27	27	27
Baseline	Mean	276.4	274.2	27	27
	SD	58.9	80.1	16.4	19.3
Visit 2 (V2)	Mean	256.7	273.3	16.1	17.7
	SD	57.2	72.1	6.1	8.2
Change at V2 from baseline	Mean	-19.7	-0.9	- 0.3	- 1.7
	SD	29.5	35.9	1.6	2.9
Visit 3 (V3)	Mean	250.4	256.4	14.9	16.8
	SD	53.0	68.3	6.1	9.2
Change at V3 from baseline	Mean	-26.0	-17.8	- 1.5	-2.6
	SD	34.6	34.4	3.0	4.0

GGTP = *gamma*-glutamyl transpeptidase; IU/l- International units per liter

**Table 4:** Levels of 1,25 dihydroxyvitamin D and Vitamin C at baseline, V2 and V3 (N = 54)

		1,25 OH Vitamin D (ng/l)		Vitamin C (μmol/l)	
		Control	PGX	Control	PGX
	N	27	27	27	27
Baseline	Mean	52.67	50.52	22.53	24.13
	SD	17.55	15.70	18.98	15.60
Visit 2	Mean	56.22	71.44	31.64	36.11
	SD	18.88	28.98	19.23	16.93
Change at V2 from baseline	Mean	3.56	20.93	9.10	11.98
	SD	20.84	24.30	15.47	14.46
Visit 3	Mean	49.67	48.26	29.16	37.22
	SD	21.67	14.11	15.19	13.72
Change at V3 from baseline	Mean	-3.00	-2.26	6.62	13.09
	SD	15.47	20.39	17.55	12.86

### Tolerance

During the study period, 8 of 27 (29.6%) subjects reported the occurrence of eleven adverse events in the control group and 9 of 27 (33.3%) subjects reported the occurrence of ten emergent adverse events in the test group. The adverse events were of mild to moderate intensity, except one severe episode of headache, unrelated to product intake. All the adverse events occurring during the study were resolved before the end of the study and required no additional follow up. Of the total number of adverse events, 14 concerned GI signs and symptoms: five occurred after intake of the control product and nine occurred after intake of test product. The relationship to study product was considered "unrelated" by the principal investigator for nine adverse events: one episode each of pharyngitis, food poisoning, rhinopharyngitis, neutropenia, and three episodes of headache (for one subject) in the control group, and one episode of headache and one episode of soft stool in the study group. The adverse events considered as "possibly" related to the use of the test product were one episode each of flatulence in the control group and the study group. One episode of nausea in the test group was considered as "probably" related to the test products intake. There were no serious adverse events reported during the study. All subjects completed the 21-day study.

## Discussion

Full agreement on the distinction between different types of dietary fiber has not been reached. However, focusing on the IOM's most recent report on the subject, *total dietary *fiber is defined as the sum of *dietary fiber *and *functional fiber*, therefore allowing for the diversity of edible, non-digestible carbohydrates and for flexibility to incorporate new fiber sources developed in the future [1].

There are large variations in the physical and chemical characteristics of dietary fiber that influence physiological responses in humans differently. Gums, which are hydrophilic polymers with significant water binding capability display unique physicochemical properties that allow them to form viscous solutions or gels when mixed with liquids. Because of these exceptional characteristics, when gums are mixed with food and human digesta in the stomach, a firm soluble polysaccharide/food matrix is formed leading to a delay in gastric emptying which was found to have several effects with possible physiological ramifications including (1) increased sensation of fullness [4], (2) possible reduction of postprandial blood glucose concentrations, potentially increasing glucose sensitivity [3], (3) delayed absorption of several nutrients in the small intestine [4,12], possibly resulting in decreased absorption of energy [13], (4) interference with the absorption of dietary fat and cholesterol with an overall result of decreased concentrations of blood cholesterol [6,14], and (5) slower transit time through the small intestine [3]. One study assessed the viscosity of different soluble and insoluble dietary fibers, and found that guar and xanthan gums had the highest viscosities, regardless of concentration and exhibited this characteristic throughout gastric and small intestinal simulation, indicating potential to elicit blood glucose and lipid attenuation [15].

In addition to the definition for dietary fiber, the amount required to impact health is also debatable. For example, IOM established an Adequate Intake (AI) recommendation for total fiber intake, based on age and sex. For adults 50 years of age and younger, the AI recommendation for total fiber intake is 38 g *per *day for men and 25 g *per *day for women, while for adults over 50 years of age, the recommendation is 30 g *per *day for men and 21 g *per *day for women. IOM did not set a Tolerable Upper Intake Level (UL) for *dietary fiber *or *functional fiber *[1]. On the other hand, FDA bases its recommendation for fiber on caloric intake. The *percent *daily value (DV) recommended by the FDA for dietary fiber for individuals consuming 2,000 kcal/day is 25 g, and 30 g for those consuming 2,500 kcal/day (21 CFR §101.9(d)(9)). However, it is evident that the intake of dietary fiber in the United States is significantly lower that the recommended amounts, as reflected by intake data from the Continuing Survey of Food Intakes by Individuals (CSFII) (1994–1996, 1998) [16] – median intakes of dietary fiber ranged from 16.5 to 17.9 g/d for men and 12.1 to 13.8 g/d for women. Based on the AI set for the various age and gender groups, 10 *percent *or less of a particular group consumed greater than the recommended AI [1].

PolyGlycopleX (PGX) is a proprietary product composed of three, highly-purified, water-soluble polysaccharides (konjac powder, sodium alginate, and xanthan gum) manufactured with the use of novel technology. The three gums act synergistically to form strong bonds that lead to a level of viscosity that is 3 to 5 times higher than any known individual polysaccharide. The final product is a soluble highly viscous *functional fiber *that can be used to provide consumers with a supplementary source of fiber in the diet.

Because of its potential use in food and dietary supplements, the present investigation was carried out to evaluate the tolerance to ingestion of 10 g PGX *per *day, for 21 days, in healthy male and female volunteers. The main objective of the study was to evaluate the overall GI tolerance, while secondary objectives were to evaluate GI signs and symptoms and assess any effects on blood hematology, biochemistry, urine and stool analysis. The protocol was developed to mimic as closely as possible a normal lifestyle and dietary intakes of healthy subjects, who did not go through a "wash out period" prior to being introduced to the test and control products. Instead, a one-week ramp-up period was used to acclimate the subjects to the new products (2.5 g BID). The maximum dose of 10 g PGX *per *day was selected based on literature values that indicate that the general population probably falls into three categories, with regards to tolerance to dietary fiber: (1) *non-sensitive *individuals, able to consume ≥ 30 g/day of fiber without experiencing undesirable gastrointestinal effects, (2) *sensitive *individuals, able to consume 10 g/day of fiber without undesirable gastrointestinal effects, but showing effects at ≥ 20 g/day, and (3) *very sensitive *individuals, who can experience undesirable gastrointestinal effects at levels of ≤ 10 g/day [17]. Also taken into consideration were the facts that dietary fibers are better tolerated if intake is in the form of solid versus liquid, and in divided versus single doses [18].

All the subjects enrolled in the trial completed the study. There were no serious adverse events and the adverse events reported were of a mild to moderate nature, and specific to GI discomfort (*e.g*., flatulence, bloating, intestinal rumbling, or abdominal pain). FDA defines a *serious adverse event *as "an adverse event that results in death, a life-threatening experience, inpatient hospitalization, a persistent or significant disability or incapacity, or a congenital anomaly or birth defect; or requires, based on reasonable medical judgment, a medical or surgical intervention to prevent an outcome described above" (Section 761(a)(2) of the FD&C Act (21 U.S.C. 379aa-1(a)(2)). An *adverse event *is defined as "any health-related event associated with the use of a dietary supplement that is adverse" (Section 761(a)(1) of the FD&C Act (21 U.S.C. 379aa-1(a)(1)). The intensity of abdominal pain and nausea were somewhat higher, but not statistically significant in the test (PGX) than in the control group, as reported in subjects' diaries. However, this was not reflected by VAS assessment. The GI complaints experienced by the subjects in this study including flatulence, bloating, abdominal distension and rumbling are well described in the literature [1], and are accepted occurrences with dietary intake of fruits and vegetables, and fiber in general [8].

There are limited studies to suggest that chronic high intakes of *dietary fiber *can cause gastrointestinal distress. The ingestion of wheat bran at levels up to 40 g/day did not result in significant increases in GI distress compared to placebo [19]. However, flatulence did increase with increased intake of *dietary fiber *in general [20], and with gums that led to moderate to severe degrees of flatulence in a trial in which 4 to 12 g/day of a hydrolyzed guar gum were provided to 16 elderly patients [21].

In the present study, none of the subjects experienced diarrhea (*diarrhea *is defined as an abnormally frequent discharge of semisolid or fluid fecal matter from the bowel, while *laxation *is a bowel movement [11]); however, the consistency of stools was softer in the test than control group at V2, although the difference was not statistically significant. The softer stools correlate with the results from the stool analysis that showed that the dry matter (%) was statistically higher in the control than in the test (PGX) group at V2. The stool softness difference was no longer present at the V3 visit, and corresponds to those documented by the IOM report indicating that viscous fiber generally has little effect on fecal bulk or laxation [1].

Statistical analysis showed no differences between test and control group with regard to urinalysis or hematological values. Although the authors believe that PGX is a fermentable fiber, the stool analysis for SCFA showed no differences between test and control groups. SCFA are highly labile and it is possible that the amount of time that lapsed between sample collection to the time of analysis was too long, possibly affecting the over all results.

The biochemical parameters were evaluated at baseline, V2 and V3 and showed no differences between test and control group, except for statistically significant decreases in total cholesterol, and LDL cholesterol at V2 and V3, and for GGTP and uric acid at V2.

PGX's effects on decreasing total and LDL cholesterol levels in the study concur with similar reports in the literature describing the effects viscous dietary fiber has on lowering serum cholesterol levels [1]. Noteworthy is the fact that not all dietary fibers decrease serum cholesterol concentration, or at least not to the same extent, with viscous fibers being most successful. Behall [22] examined the effects a low- and high-fiber diet had on plasma cholesterol concentrations. The high-fiber diet of an average of 19.5 g/day was further divided into non-viscous fiber (cellulose), and viscous fiber (carboxymethylcellulose gum, karaya gum, or locust bean gum). Results showed that diets containing viscous dietary fiber led to significantly lower plasma cholesterol concentrations. Jenkins *et al*. [23] reported the hypocholesterolemic effect of guar gum in the mid 1970s, followed in the 1980s by other studies that showed a significant reduction in serum cholesterol concentrations of between 11 and 16 *percent *[23-25]. Anderson *et al *[26] demonstrated that when 20 hypercholesterolemic men were randomly assigned to either a wheat bran or oat bran diet, a significant decrease in serum total cholesterol concentration of 12.8 *percent *was noted only after 21 days; however, this effect was not seen in the wheat bran group.

Two *meta*-analyses evaluating the effects of viscous fiber arrived at the same conclusions. One *meta*-analysis of 20 trials that used high doses of the viscous fiber oat bran, showed that the reductions in serum cholesterol concentrations ranged from 0.1 to 2.5 *percent*/g of intake [27], while another, evaluating the effects of oat bran, pectin, psyllium and guar gum on blood lipid concentrations showed that 2 to 10 g/day of viscous fiber were associated with small but significant decreases in total and LDL cholesterol concentrations [28].

IOM also concluded that viscous *dietary *and *functional fibers *reduce both total and LDL cholesterol concentrations, and possibly serum triglycerides in a dose-dependent manner, although the report acknowledges that only few studies report dose-response data. Further, the report indicates that these relatively small-scale intervention trials using viscous *functional fibers *have reported substantial cholesterol-lowering effects and therefore, probably have protective effects against coronary heart disease (CHD) [1].

The GGTP levels were statistically lower in the PGX group compared to controls. While the exact mechanism that led to this decrease is not fully understood, especially because the other liver enzymes were not altered, it is the belief of the investigators that this decrease is beneficial, as elevation in GGTP is used to assess possible damage to the liver or the biliary system.

Elevated levels of uric acid are associated with gout and recently have been considered important markers for hypertension, cardiovascular disease and metabolic syndrome [29]. While most studies have focused on hyperuricemic states caused by either over production or under excretion of uric cid, as well as by excessive intake of ribonucleic acid (RNA), few studies have been devoted to hypourecemia, or the effects of maintaining low uric acid levels in otherwise healthy individuals [30]. Specifically, the effect of dietary fiber on serum uric acid levels has been only minimally investigated. Koguchi *et al*. [31] have shown that feeding rats a diet high in viscous fiber (*e.g*., xanthan gum) they were able to alter metabolic processes that contribute to hyperurecemia by lowering serum uric acid levels, reducing RNA digestion and increasing RNA excretion in the feces [31], a mechanism that probably also explains the statistically lower levels of serum uric acid identified in this clinical study at the V2 visit.

Lastly, the serum levels of several fat- and water soluble vitamins were assessed during the study. Vitamin D was measured because PGX demonstrates specific physical properties including high viscosity and solubility and, there was concern for possible sequestration of fat soluble vitamins and therefore vitamin malabsorption. Consequently, vitamin A, D, E and K levels were monitored during the study. Vitamin C and B levels were followed as an assessment of the potential of PGX to result in generalized malabsorption. Results showed that there were no differences observed in the levels of vitamin A, B_1_, B_6_, B_12_, E, and K between test and control group, except for statistically higher levels of plasma vitamin D (1,25-dihydroxyvitamin D) and C. Vitamin D is a fat soluble vitamin found only in few foods and produced endogenously with exposure to sunlight. The vitamin D ingested or produced is inactive (25-hydroxyvitamin D [25(OH)D] (calcidiol)) and undergoes activation through a two-step hydroxylation process in the liver and kidneys to form 1,25-dihydroxyvitamin D [1,25(OH)_2_D] (calcitriol). According to the National Institutes of Health/Office of Dietary Supplements (NIH/ODS), circulating 1,25(OH)_2_D is generally not a good indicator of vitamin D status, because it has a short half-life of 15 hours and serum concentrations are closely regulated by calcium, phosphate and parathyroid hormone. Further, levels of 1,25(OH)_2_D do not decrease until vitamin D deficiency is severe [32]. Adequate levels of vitamin D are essential for bone and overall health. However, it has been determined that 41 *percent *of American men and 53 *percent *of American women have levels of vitamin D below what is considered optimum. A recent study found that a low serum level of 25-hydroxyvitamin D could be independently associated with a significantly increased risk of all-cause mortality. According to the authors, if these findings are confirmed in future clinical studies, vitamin D supplementation should be studied as a way to reduce mortality risk [33].

Interestingly, optimal serum concentrations of 25(OH)D have not been established [34,35] and are likely to vary at each stage of life. NIH considers a concentration of <50 nmol/L 25(OH)D generally inadequate. In 2007, a controversial editorial was published contending that supplemental intakes of 400 International Units (IU)/day of vitamin D increase 25(OH)D concentrations by only 2.8–4.8 ng/mL (7–12 nmol/L) and that daily intakes of approximately 1,700 IU are needed to raise these concentrations from 20 to 32 ng/mL (50 to 80 nmol/L) [36]. The exact mechanism leading to higher levels of serum 1,25(OH)_2_D in our study is not completely understood, or interpreted as a good indicator of vitamin D status. However, based on evidence that subnormal levels of vitamin D can impact bone and overall health, and that according to some investigators, current recommendations for supplementation of the US population is approximately 4-fold lower than it should be, it is likely that consumers could benefit from increased levels of serum vitamin D to provided the necessary health benefits.

The exact mechanism causing increase in the serum vitamin C levels in the test group is not completely understood; however, as a water soluble vitamin, the somewhat higher levels are not anticipated to have any detrimental effects in otherwise healthy subjects.

The effects of the intervention on dietary intakes were not measured in this clinical trial, because the focus of the study was on assessing GI tolerance. However, this intentional omission could be interpreted as a limitation of the study, which the authors plan in addressing in future clinical trials.

## Conclusion

PGX, a novel, soluble and highly viscous *functional fiber *showed in clinical testing to be well tolerated by healthy male and female subjects when used to supplement the diet for up to 10 g *per *day. Because the intake of dietary fiber is significantly lower that the recommended amount for the US population, the addition of PGX to a regular diet will benefit consumers by increasing the overall fiber intake; and because of its unique composition generating a 3 to 5 times higher viscosity that any single polysaccharide, this *functional fiber *can also provide consumers with added physiological benefits including, but not limited to maintenance of healthy total and LDL cholesterol and uric acid levels.

## Competing interests

All authors have a financial relationship with the sponsor of the study, InovoBiologic, Inc., Calgary, Alberta, Canada.

## Authors' contributions

IC participated in the design of the clinical trial protocol, review of data, drafting and finalizing the manuscript. ML has been involved in evaluating the physiological effects of the test substance and participated in developing the protocol. SW provided input for the clinical trial protocol and manuscript. XP developed the clinical trial protocol and conducted the study. YD developed the clinical trial protocol and conducted the study. GB provided input for the clinical trial protocol and edited the manuscript. All authors read and approved the final manuscript.

## Supplementary Material

Additional File 1 **Appendix I.** Assay methods employed for biochemical, hematological and urine analysis. The data provided represents assay methods used for testing. Click here for file
