# Supplementary material for: Supplementation of the diet with the functional fiber PolyGlycoplex® is well tolerated by healthy subjects in a clinical trial
Source: Nutr J. 2009 Feb 5;8:9. doi: 10.1186/1475-2891-8-9 (PMC2657159; doi:10.1186/1475-2891-8-9)
Supplement: Additional File 1 — Appendix I. Assay methods employed for biochemical, hematological and urine analysis. The data provided represents assay methods used for testing. [file 1475-2891-8-9-S1.doc]

APPENDIX I

| **Assay methods employed for biochemical, hematological and urine analysis** | |
| --- | --- |
| **BIOCHEMISTRY**  Conducted with ROCHE/HITACHI 912, Roche Diagnostics, Inc. |  |
| Bun/Urea | UV kinetic test. |
| Creatinine | JAFFE method with compensation. Kinetic colorimetric test. |
| Total protein | BIURET method. Colorimetric test. |
| Total bilirubin | JENDRASSIK GROF method. Colorimetric method. |
| Glucose | Glucose GOD-PAP method. Enzymatic colorimetric method. |
| Total cholesterol | Enzymatic colorimetric method. |
| HDL cholesterol | Enzymatic colorimetric test in homogenous phase. |
| LDL cholesterol | Enzymatic colorimetric test in homogenous phase. |
| Triglycerides | Enzymatic colorimetric TRINDER modified final point method. |
| Alkaline phosphatase | Enzymatic kinetic method |
| Alanine amino transferase. ALAT/GPT/TGP/ALT | IFCC method. Enzymatic kinetic. |
| Aspartate amino transferase. AST/ASAT/GOT/TGO | IFCC method. Enzymatic kinetic. |
| GGT | SZASZ method (kinetic photometric method). Colorimetric method |
| LDH | “Optimised method” according to the Germany Society of Clinical Chemistry. |
| CPK | “Optimised method” according to the IFCC. |
| Sodium, potassium, chloride | Indirect potentiometric analysis |
| Calcium | Colorimetric test. Final point method with reagent control test |
| Uric acid | Enzymatic colorimetric method |
| Albumin | Immunoturbidimetric analysis |
| Magnesium | Colorimetric test using a final point method for the quantitative determination of magnesium in serum or plasma |
| Ferritin | Immunoenzymological microparticular MEIA technique |
| **HEMATOLOGY**  Conducted with HORIBA ABX PENTRA 120 DX. Horiba Medical Diagnostics, Ltd. | The results for RBC, WBC, platelet, basophil counts, and the level of hemoglobin and hematocrit were measured.  The results for MCV, MCH, MCHC were obtained through calculations using computer software.  The differentiation of the blood elements is based on physical (light diffraction), and chemical principles (affinity for a colour) which are different for each population. |
| **TOXICOLOGY/SEROLOGY/HORMONES**  Conducted on AxSYM® ABBOTT DIAGNOSTICS |  |
| Zinc (Pasteur Cerba Laboratory) | Atomic Absorption Spectrometry technique |
| Vitamin A (Pasteur Cerba Laboratory) | HPLC technique |
| Vitamin B1 (Pasteur Cerba Laboratory) | HPLC technique |
| Vitamin B6 (Pasteur Cerba Laboratory) | HPLC tecnique |
| Vitamin B12 (Pasteur Cerba Laboratory) | Chemiluminescence technique |
| Vitamin C (Pasteur Cerba Laboratory) | HPLC technique |
| Vitamin D (1.25 OH) (Pasteur Cerba Laboratory) | Radioimmunological technique |
| Vitamin E (Pasteur Cerba Laboratory) | HPLC technique |
| Vitamin K (Pasteur Cerba Laboratory) | HPLC technique |
| TSH  - Conducted on BAYER CENTAUR MACHINE - | Immunoenzymological ELISA third generation technique |
| **URINE ANALYSIS**  Performed with COMBUR-Test® strips, ROCHE DIAGNOSTICS | Tested urinary pH, glucose, protein, blood & ketone bodies |
| The functioning of the PENTRA 120 DX, HITACHI 912 ROCHE DIAGNOSTICS and AXSYM ABBOTT conform to the norms of the ISO 9002 certification.  HPLC – high performance liquid chromatography  IFCC – International Federation of Clinical Chemistry  ELISA - enzyme-linked immunosorbent assay  HORIBA ABX Diagnostics is a division of HORIBA Ltd., Kyoto, Japan  Roche Diagnostics Combur-Test® strips, in the USA and Canada are known as Chemstrips | |
